# The Exon Junction Complex Factor RBM8A in Glial Fibrillary Acid Protein-Expressing Astrocytes Modulates Locomotion Behaviors

**DOI:** 10.3390/cells13060498

**Published:** 2024-03-13

**Authors:** Shravan Asthana, Jennifer Mott, Mabel Tong, Zifei Pei, Yingwei Mao

**Affiliations:** 1 Department of Biology, Pennsylvania State University, University Park, PA 16802, USA; shravanasthana@gmail.com (S.A.); jbm6671@psu.edu (J.M.); mst5405@psu.edu (M.T.); zzp1@psu.edu (Z.P.); 2 Feinberg School of Medicine, Northwestern University, 303 East Superior Street, Chicago, IL 60611, USA

**Keywords:** exon junction complex, RBM8A, astrocyte

## Abstract

The role of RNA Binding Motif Protein 8a (RBM8A), an exon junction complex (EJC) component, in neurodevelopmental disorders has been increasingly studied for its crucial role in regulating multiple levels of gene expression. It regulates mRNA splicing, translation, and mRNA degradation and influences embryonic development. RBM8A protein is expressed in both neurons and astrocytes, but little is known about RBM8A’s specific role in glial fibrillary acid protein (GFAP)-positive astrocytes. To address the role of RBM8A in astrocytes, we generated a conditional heterozygous knockout (KO) mouse line of *Rbm8a* in astrocytes using a GFAP-cre line. We confirmed a decreased expression of RBM8A in astrocytes of heterozygous conditional KO mice via RT-PCR and Sanger sequencing, as well as qRT-PCR, immunohistochemistry, and Western blot. Interestingly, these mice exhibit significantly increased movement and mobility, alongside sex-specific altered anxiety in the open field test (OFT) and elevated plus maze (OPM) tests. These tests, along with the rotarod test, suggest that these mice have normal motor coordination but hyperactive phenotypes. In addition, the haploinsufficiency of *Rbm8a* in astrocytes leads to a sex-specific change in astrocyte density in the dentate gyrus. This study further reveals the contribution of *Rbm8a* deletion to CNS pathology, generating more insights via the glial lens of an *Rbm8a* model of neurodevelopmental disorder.

## 1. Introduction

Neurodevelopmental disorders are a collection of disabilities and impairments related to central nervous system (CNS) dysfunction in motor, cognition, communication, interaction, and emotion, including autism spectrum disorders (ASDs), intellectual disability (ID), and attention-deficit/hyperactivity disorder (ADHD). Neurodevelopmental disorders affect more than 3% of children worldwide and the comorbidity of conditions is often observed [1]. A defining characteristic of these disorders is their onset before puberty in a uniquely steady course. For example, the impaired social-communication functions and restricted, repetitive pattern of behaviors can be detected in children with ASDs at an age of 2–3 years. As individuals with neurodevelopmental disorders continue into adulthood, outcomes can be highly variable both across and within disorders. Neurodevelopmental disorders are highly multifactorial, with both a tremendous genetic and environmental influence in their origin, as well as their heterogeneity in clinical presentation and treatment [2].

The EJC is composed of RBM8A, Mago Homolog (MAGOH), Eukaryotic Translation Initiation Factor 4A3 (EIF4A3), and Metastatic Lymph Node 51 (MLN51). EJC formation is activity-dependent and functions to directly control mRNA splicing, export, translation, and degradation [3,4,5,6,7]. The EJC plays a significant role in nonsense-mediated mRNA decay (NMD), a well-conserved RNA surveillance mechanism, which identifies and degrades aberrant mRNAs carrying premature termination codons (PTCs) [8]. Dysfunctions in several NMD and EJC factors have been implicated in various human diseases [9,10,11,12], including ASDs [13], ID [14,15,16,17], schizophrenia [13,18], Thrombocytopenia with Absent Radius syndrome (TAR) [19,20], developmental delay [21], and Richieri–Costa–Pereira syndrome [22]. In humans, the deletion of 1q21.1 and single-nucleotide polymorphisms (SNPs) in the *RBM8A* noncoding regions results in TAR—a blood and limb disorder—in addition to neurodevelopmental phenotypes at an increased incidence [19,20].

We have previously shown that RBM8A is critical for the proper proliferation and differentiation of cortical neural progenitor cells (NPCs) [23,24]. The overexpression of RBM8A in vivo stimulated embryonic NPC proliferation and suppressed neuronal differentiation. *Rbm8a* knockdown in the embryonic cortex reduced NPC proliferation and promoted premature neuronal differentiation [23]. *Rbm8a* was shown to be necessary to regulate the NPC pool in both the subventricular zone (SVZ) and ventricular zone (VZ) by acting on cell cycle progression and the differentiation of NPCs [23]. RBM8A-deficient cells have also been found to accumulate DNA damage, which decreased the viability and proliferation of NPCs [25]. *Rbm8a* mRNA is regulated by other factors, such as the microRNA miR-29a in retinal progenitor cells [26]. miR-29 is capable of repressing *Rbm8a* by binding to the 3′-UTR, which upregulates differentiation and downregulates proliferation [26]. RBM8A depletion in human A549 tumor cells results in apoptosis, supporting the critical role of MAGOH and RBM8A in proper mitotic phase progression, which can serve as a potential anticancer therapeutic target [27]. Consistently, we have previously reported that RBM8A haploinsufficiency significantly altered the distribution of interneurons in the mouse cortex [24]. Taken together, these results support the key role of the EJC in neurodevelopment.

Astrocytes and microglia are known to be in close communication with neurons throughout development. Astrocytes, in particular, are able to engage in bi-directional signaling with thousands of synapses to form tripartite synapses [28]. Astrocytes exhibit tremendous diversity in electrophysiology, the transcriptome, and proteome, as well as morphology in different neural regions [29]. Many studies have revealed the different roles of the astrocyte in metabolism regulation, energy supply, neural specific factor secretion, and gliotransmission [28]. Both astrocyte secretion and contact are involved in synapse formation, maturation, functionalization, stabilization, and even pruning [30]. The elaboration of the fine astrocyte processes involved in the tripartite synapse may not entirely take place during the large generation of astrocytes before birth, but instead may coincide with the active period of synaptogenesis.

Astrocytes play an important role in the inflammatory and immune response of the nervous system. A postmortem analysis of human brains with ASDs demonstrates increases in glial reactivity, suggesting that the dysregulation of an astrocyte-mediated inflammatory response may be involved in the generation of ASD brains [28]. Genomic analyses of these patients revealed many ASD candidate genes are enriched in astrocytes [28]. In a Rett syndrome (RTT) model with astrocyte-specific *MeCP2* KO, neurons showed decreased dendritic arborization, spine plasticity, and reduced cortical plasticity [31,32]. Interestingly, in a Fragile X Syndrome (FXS) mouse model, the FMRP loss of function in astrocytes led to a decreased survival, decreased synaptic protein clustering at both the pre and post synaptic membrane, and stunted dendritic arborization [33,34,35,36]. Astrocytes and microglial dysfunction have also been implicated in the pathogenesis of neurodegenerative diseases and in RTT and Parkinson’s disease; astrocytes may be able to independently cause the disease phenotype in the mouse [34].

Manipulation of the RBM8A level in the dentate gyrus neurons causes a change in behavioral pattern [37]. However, the role of RBM8A in astrocytes related to behavioral regulation has not been explored. Given the implication of RBM8A in several neurodevelopmental disorders, as well as the critical role of astrocytes in their pathogenesis, we explored how *Rbm8a* haploinsufficiency in astrocytes modulates animal behaviors using a novel mouse model carrying conditional heterozygous deletion of the *Rbm8a* gene, which may provide some insights into the role of astrocytes in neurodevelopmental disorders.

## 2. Materials and Methods

### 2.1. Generation of the GFAPCre;Rbm8a^f/+^ Mice

*Rbm8a*-floxed mice (*Rbm8a^f/f^*) without the Neo cassette were previously generated in our lab [24]. Breeding approaches were performed as follows: The *Rbm8a^f/f^* mice were crossed with female F1 offspring from a commercial F0 hemizygous JAX #012886 B6.Cg-Tg(Gfap-cre)73.12Mvs/J congenic/transgenic model (The Jackson Lab, Bar Harbor, ME, USA). All the female offspring heterozygously express the transgenic glial fibrillary acid protein (GFAP) promoter-driven expression of Cre recombinase (GFAPCre). With this approach, independently of the sexes, we selectively obtained *Rbm8a* haploinsufficiency in astrocytes in half of the offspring (GFAPCre; *Rbm8a^f/^*^+^) or, as control condition, *Rbm8a^f/+^*, expected in the progeny at a rate of 50%. However, we cannot detect GFAPCre; *Rbm8a^f/^*^f^ mice in the progenies at postnatal stage P15, suggesting the lethality of GFAPCre; *Rbm8a^f/^*^f^ mice at either the embryonic or early neonatal stages.

### 2.2. Animals

All procedures on mice were reviewed and approved by The Pennsylvania State University IACUC committee, under IACUC protocols to Yingwei Mao. All mice were housed by sex, with 2–4 mice per cage in a room with a light/dark cycle of 12 h intervals (lights on at 7:00 am) and were provided with ad libitum access to food and water. Mouse behavior tests were performed in animals only after reaching 60 days after birth. Behavior testing ceased at 120 days after birth. For all behavior tests, 24 GFAPCre; *Rbm8a^f/+^* mice (10 female, 12 male) and 23 *Rbm8a^f/+^* mice (11 female, 12 male) were used. Mice at 5–6 months of age were anesthetized with 2.5% avertin and were perfused with 4% paraformaldehyde (PFA) in PBS for additional analyses.

### 2.3. Immunohistochemistry

The brains were fixed in 4% PFA for 24 h at 4 °C, then transferred to PBS for storage. The brains were sagittally sectioned at 40 µm with a vibratome. Immunohistochemistry was performed to stain specific proteins in the tissue slices, selecting for antibodies against RBM8A (cat#GTX131387, GeneTex, Irvine, CA, USA), astrocyte marker GFAP (cat#AB5541, Millipore Sigma, Billerica, MA, USA), and neuronal marker NeuN (cat#ABN90P, Millipore Sigma, St. Louis, MO, USA), according to previous studies [24]. Three GFAPCre; *Rbm8a^f/+^* mice and three *Rbm8a^f/+^* mice were used. Cell fluorescence intensity was measured using ZEISS Zen software. These images were further analyzed using FIJI/ImageJ version 2.14.0/1.54f. The hippocampus area was analyzed using the “analyze particle” feature set to 0.5 μm^2^–infinity. The scale was set to 3.16 pixels/μm. Astrocyte density was calculated as the number of cells in a set area.

### 2.4. RNA Isolation

Upon sacrifice, the brains were flash frozen using liquid nitrogen, then stored at −80 °C. The brain hemisphere was used to isolate the RNA using TRIzol^TM^ reagent, following manufacturer instructions. Three GFAPCre; *Rbm8a^f/+^* mice and three *Rbm8a^f/+^* mice were used.

### 2.5. Quantitative Reverse Transcription–PCR (qRT-PCR)

**Figure 1:**
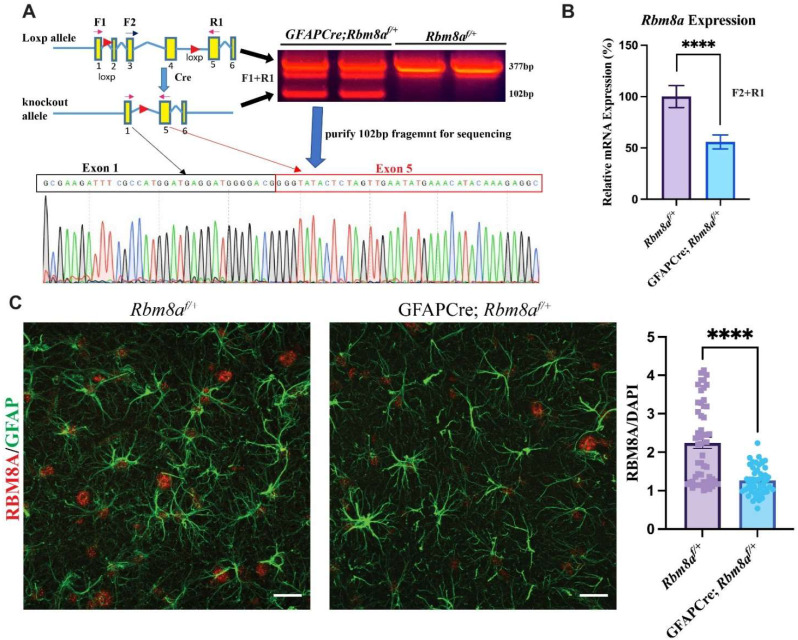
Confirmation of reduced *Rbm8a* levels in GFAPCre; *Rbm8a^f/+^* mice. (**A**) RT-PCR using primers F1 and R1 outside of loxp sites detected the predicted deletion 102 bp band. Sequencing result of the 102 bp fragment confirmed the joining of exon1 and exon 5, lacking exons 2–4 in the deleted *Rbm8a* mRNA from the GFAPCre;*Rbm8a^f/+^* mouse brain. (**B**) Validation of conditional KO mouse model using qRT-PCR. Relative *Rbm8a* mRNA levels in the GFAPCre;*Rbm8a^f/+^* mouse brain are about 55.85% of those in the control brain. Data shown are mean +/− SEM of 8 runs of qPCR from *Rbm8a^f/+^* and GFAPCre; *Rbm8a^f/+^* mice (n = 3 each group). ****, *p* < 0.0001. (**C**) Expression of RBM8A (red) in astrocytes (GFAP; green) in the hippocampus of sagittal sections of *Rbm8a^f/+^* mice and GFAPCre; *Rbm8a^f/+^* mice. Bar graph shows immunofluorescence intensity of RBM8A, normalized by that of DAPI. Data shown are mean +/− SEM of more than 60 astrocytes from *Rbm8a^f/+^* and GFAPCre; *Rbm8a^f/+^* mice (n = 3 each group). ****, *p* < 0.0001. Scale bar = 20 µm.

The total RNAs were converted into cDNA by reverse transcription with the oligo-dT primer, using Superscript reverse transcriptase III (Invitrogen, Catalog number: 18080093). RT-PCR to amplify the deletion fragment used primers F1 (5′-GCGAAGATTTCGCCATGGAT-3′) in exon 1 and R1 (5′- TTGACCATTTAGTCCTTCCA-3′) in exon 5, outside of loxp sites in the cKO genome (Figure 1A). The cDNA fragment with exon 2-4 deletion was gel purified and submitted for Sanger sequencing using R1 primer in the PSU Genomic Core facility. qRT-PCR was performed using PerfeCTa SYBR Green SuperMix (Quantabio, Beverly, MA, USA) and mouse RBM8A primers (F2: 5′-ATTACGACAGTGTGGAGCAG-3′, in exon 3, and R1). The expression of the β-Actin gene (forward: 5′- CGTGGGCCGCCCTAGGCACCA-3′, reverse: 5′-TTGGCCTTAGGGTTCAGG GGGG-3′, IDT) was used as an internal control. The qPCR reactions were performed on a StepOnePlus Real-Time PCR system (Applied Biosystems, Carlsbad, CA, USA) and the Ct calculations were performed using StepOne software version 2.3.

### 2.6. Western Blot

The total protein samples isolated from mouse brains were resolved by 10% SDS–polyacrylamide gel electrophoresis and were subsequently transferred to nitrocellulose membranes. The blots were blocked with 5% milk in TBST (10 mM Tris-HCl, pH 8.0, 150 mM NaCl, and 0.5% Triton X-100) for 1 hour at room temperature. Blots were then incubated overnight with rabbit anti-RBM8A (cat#GTX131387, GeneTex, Irvine, CA, USA, 1:1000) and mouse anti-Actin antibody (cat#sc-8432, Santa Cruz Biotechnology, Dallas, TX, USA, 1:250) in 5% milk in TBST at 4 °C, with shaking. Donkey anti-rabbit IgG (cat#926-68023, LI-COR, Lincoln, NE, USA, 1:20,000) and donkey anti-mouse IgG (cat#926-32212, LI-COR, Lincoln, NE, USA, 1:20,000) were used as secondary antibodies. Immunoreactivity was detected using the LI-COR Odyssey imaging system, according to the manufacturer’s instructions.

### 2.7. Open Field Test (OFT)

The open field apparatus is a versatile paradigm utilized to investigate motor, anxiety, and stress behaviors in rodents [38]. The test was performed as described in [39], with no changes. The mice were randomly placed in an acrylic white box (40 × 40 × 40 cm) in a room brightly lit, with no shadows in the box. The EthoVision XT 9 software by Noldus [40] recorded horizontal movement for five minutes. Several measures were taken regarding the distance traveled, the average velocity, the duration in the zone, and the frequency of the zone throughout the entire box (40 × 40 cm) versus the center zone (13.3 × 13.3 cm). In addition, 70% ethanol was used to clean the apparatus between each trial.

### 2.8. Rotarod

The rotarod apparatus is used to assess maximal motor skill and motor memory [41]. Here, it was used to evaluate potential deficits in motor coordination. The test was performed as described in [39]. The mice were placed on a rotating platform starting at four revolutions per minute (rpm). Over the course of five minutes, the platform accelerated to 40 rpm. Then, the platform was set to maintain this speed for an additional five minutes. The time at which the mouse fell from the platform was recorded as the falling time. The procedure was repeated for three trials per mouse, per day, for three consecutive days. One week after the first test day, the final test was performed. Additionally, 70% ethanol was used to clean the apparatus between each trial.

### 2.9. Tail Suspension

The tail suspension test was used to assess the depressive affect by measuring the immobility time. The test was performed as described in [39], with no changes. Mice tails were affixed to a rod 60 cm above the ground. Immobility time—defined as the duration where the mouse remained still—was measured manually with a stopwatch.

### 2.10. Elevated Plus Maze (EPM)

The elevated plus maze apparatus is a versatile paradigm utilized to investigate motor and anxiety behavior in rodents [42]. The test was performed as described in [39], with no changes. Mice were randomly placed into the crossing region (5 × 5 cm) of an acrylic platform, 50 cm above the ground, with arms (5 × 25 cm) either enclosed in walls or open to the environment. The Noldus EthoVision XT software [40] recorded horizontal movement for ten minutes. Several measures were taken regarding the distance traveled, average velocity, duration in arms, and arm frequency. Additionally, 70% ethanol was used to clean the apparatus between each trial.

### 2.11. Marble Burying

The marble burying test is used to assess repetitive and obsessive behaviors. The test was performed as described with no changes. The mice were placed in empty cages except for 3 cm of bedding and four rows of five clear marbles. After ten minutes, the number of buried marbles (defined as being at least 2/3 obscured) was measured.

### 2.12. Nest Building

The nest building test is also used to assess repetitive and obsessive behaviors. The test was performed as described in [39], with no changes. Mice were placed into empty cages except for 1 cm of bedding and a fresh 2 g nestlet. Photographs were taken at both one hour and 24 h after the test began. The images were assessed by three independent observers and scored (1–5) according to criteria described in [43].

### 2.13. Statistical Analysis

Statistical analysis of these behavioral datasets took the form of Excel, SPSS, and GraphPad PRISM 10 software, expressed as means +/− standard error of means (SEM). The normality of the distributions was assessed by using both the Kolmogorov–Smirnov and Shapiro–Wilk tests. For those distributions that did not reach normality, a Mann–Whitney U test was used to assess the differences between the means of the groups to assess statistical significance. Otherwise, Student’s *t*-tests were used. Statistical significance was evaluated at an alpha = 0.05 level, irrespective of the test used.

## 3. Results

### 3.1. Validation of GFAPCre; Rbm8a^f/+^ Mouse Model

We developed an *Rbm8a^f/f^* mouse model with loxp sites flanking exons 2, 3, and 4 (Figure 1A) [24]. Because of the early lethality of GFAPCre; *Rbm8a^f/f^* mice, we focused on studying the phenotypes of heterozygous GFAPCre; *Rbm8a^f/+^* mice. We first confirmed the deletion of exons 2–4 in mRNA isolated from the brain of a GFAPCre; *Rbm8a^f/+^* mouse using forward primer F1 in exon 1 and reverse primer R1 in exon 5 (Figure 1A), which are localized outside of loxp sites. RT-PCR detected a predicted deletion band at 102bp, lacking exon 2–4, compared to a normal 377 bp band from control *Rbm8a^f/+^* mice (Figure 1A). The 102 bp deletion fragment was purified for Sanger sequencing. The results confirmed the joining of exon 1 and exon 5 in the *Rbm8a* transcript (Figure 1A), supporting the fact that Cre recombinase successfully deleted exons 2–4 of *Rbm8a* mRNA in astrocytes of GFAPCre; *Rbm8a^f/+^* mice. Although we can detect the mutant band in Figure 1A, it is expressed at an extremely low level compared to the WT transcript, suggesting a rapid degradation of the truncated transcript.

To further measure the mRNA level of remaining normal *Rbm8a* transcript in our conditional knockout mouse model, we performed qRT-PCR using primers F2 and R1 (Figure 1B). F2 is localized in exon 3, which will be removed by Cre (Figure 1A,B) in the astrocytes of GFAPCre; *Rbm8a^f/+^* mice. Therefore, F2 + R1 PCR only detects the normal *Rbm8a* transcript. *Rbm8a* expression levels in the GFAPCre; *Rbm8a^f/+^* mouse brain were 55.85% of that in the *Rbm8a*^*f*/^mouse brain, confirming a reduction in gene expression. We detected about half of the *Rbm8a* transcript in the brain of GFAPCre; *Rbm8a^f/+^* mice.

To examine RBM8A protein level in GFAPCre; *Rbm8a^f/+^* mice, immunohistochemistry on sagittal sections of control mice and GFAPCre; *Rbm8a^f/+^* mice at six months was performed for RBM8A and GFAP (Figure 1C). Consistently, compared to control mice, RBM8A expression was noticeably reduced in GFAPCre; *Rbm8a^f/+^* mice in the hippocampus. When the immunofluorescence intensity of RBM8A was normalized to that of DAPI staining, the quantification showed RBM8A expression is significantly reduced, by about half, in *GFAPCre; Rbm8a^f/+^* mice compared to *Rbm8a^f/+^* mice (Figure 1C). Furthermore, a Western blot using brain lysates from control and GFAPCre; *Rbm8a^f/+^* mice confirmed decreased RBM8A protein levels (Supplemental Figure S1A). The RBM8A protein levels in neurons of the cortex and hippocampus were not changed (Supplemental Figure S1B–D).

Taken together, our sequencing, qRT-PCR, immunohistochemistry, and WB analyses validated that our conditional heterozygous KO mice show the expected decreased expression of RBM8A in astrocytes.

### 3.2. GFAPCre;Rbm8a^f/+^ Mice Show Abnormal Locomotion Activity in the OFT

**Figure 2:**
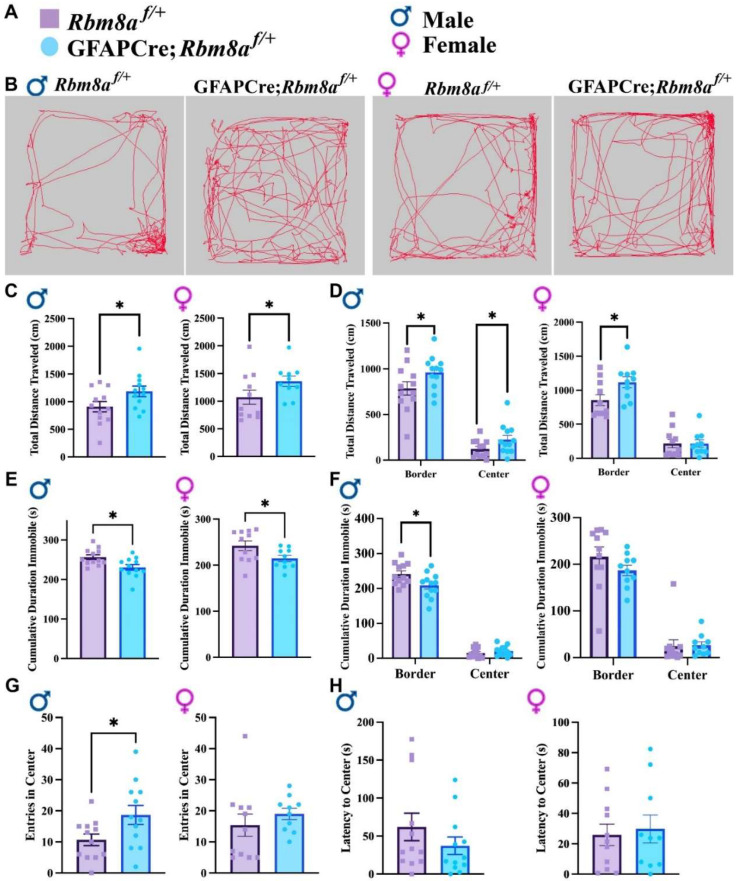
Results of the OFT across genotypes and sexes. (**A**) Figure legend for genotypes and sexes. (**B**) Representative track of mouse movement in the OFT. (**C**) Total distance traveled in the OFT. (**D**) Total distance traveled on the border versus center of the open field. (**E**) Cumulative duration immobile in the OFT. (**F**) Cumulative duration immobile on the border and center of the open field. (**G**) Frequency of entry in the center. (**H**) Latency to the center zone. Data shown are mean +/− SEM for control mice *Rbm8a^f/+^* (N = 21, male = 12, female = 11) and experimental mice GFAPCre; *Rbm8a^f/+^* (N = 22, male = 12, female = 10), where * indicates a *p*-value < 0.05.

To determine how the haploinsufficiency of *Rbm8a* in astrocytes affects overall animal behaviors, total distance traveled was measured to track cumulative movement across the arena using EthoVision [40]. EthoVision software can specifically measure the distance moved from the center point of the mouse. We first tested the mice in the OFT, which allowed us to measure the locomotion and anxiety-like behaviors (Figure 2A,B). Interestingly, both male (*p* = 0.049) and female (*p* = 0.045) GFAPCre; *Rbm8a^f/+^* mice exhibit a significantly increased total distance traveled, respectively, compared to the control group (Figure 2C). Consistent with increased locomotion, both male (*p* = 0.011) and female (*p* = 0.045) experimental mice demonstrate a decreased cumulative duration of immobility (Figure 2E). When we further tracked in which zone the mice traveled more, we started to detect interesting sex differences (Figure 2D,F). Male GFAPCre; *Rbm8a^f/+^* mice exhibited a significantly higher distance traveled in both the border (*p* = 0.034) and the center (*p* = 0.04), whereas females (*p* = 0.032) only showed higher locomotion in the border area (Figure 2D). Consistently, less immobile time spent in the border zone was detected only in male GFAPCre; *Rbm8a^f/+^* mice (*p* = 0.026) but not in females (Figure 2F), suggesting a sex-specific difference in zone preference.

In the OFT, mice are expected to prefer hiding in the corners or border of the acrylic apparatus, reflecting a native tendency to avoid predators. An abnormal interaction or affinity with the center of the testing arena, an exposed zone, is indicative of an altered anxiety state [38], which can be measured by cumulative travel or duration in the center, as well as the frequency and latency to enter the center. Interestingly, in the OFT, male but not female GFAPCre; *Rbm8a^f/+^* mice traveled longer distances in the center (Figure 2D). Consistently, male GFAPCre; *Rbm8a^f/+^* mice (*p* = 0.036) demonstrate a significantly higher frequency to the center (Figure 2G), although both male and female GFAPCre; *Rbm8a^f/+^* mice are not statistically distinct from control mice in relation to the cumulative duration in the center or the latency to the center (Figure 2H). A slight decrease in the anxiety-like behavior was measured exclusively in male mice, prompting us to further analyze the anxiety-like phenotype in *Rbm8a* mice with other experimental tools, as the hyperactivity could be a confounding factor for an anxiety-like phenotype. We opted for the elevated plus maze (EPM) test as a suitable approach–avoidance test, frequently used as a proxy to evaluate anxiety in rodents.

Overall, it is then clear in the OFT that experimental mice traveled more and displayed an increased locomotion. These data suggest a hyperactive phenotype in which GFAPCre; *Rbm8a^f/+^* mice are generally more active in both distance and duration.

### 3.3. Altered Behavior in GFAPCre; Rbm8a^f/+^ Mice in the EPM Test

**Figure 3:**
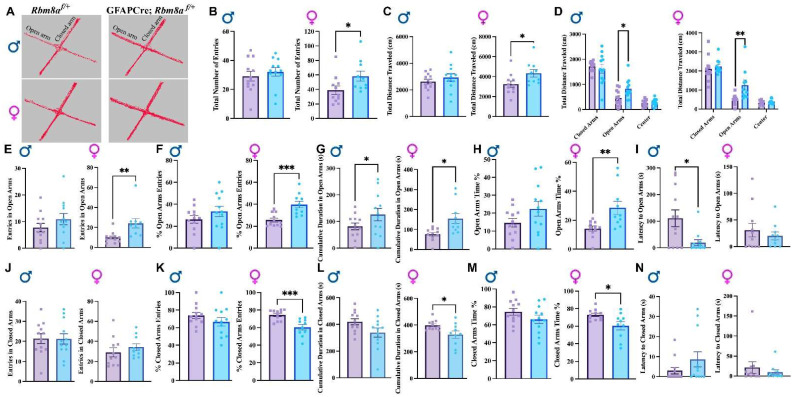
EPM test results support reduced anxiety-like phenotypes. (**A**) Representative track of female mouse movement in the EPM test. (**B**) Total arm entries in the EPM test. (**C**) Total distance traveled in the EPM test. (**D**) Total distance traveled in the closed arms, open arms, and center of the EPM test. (**E**) Total entries to the open arms. (**F**) Percentage of open arm entries in the EPM test. (**G**) Cumulative duration in open arms. (**H**) Percentage of time spent in open arms. (**I**) Latency to the open arms. (**J**) Total entries to the closed arms. (**K**) Percentage of closed arm entries in the EPM test. (**L**) Cumulative duration in closed arms. (**M**) Percentage of time spent in closed arms. (**N**) Latency to the closed arms. Data shown are mean +/− SEM for control mice *Rbm8a^f/+^* (N = 21, male = 12, female = 11) and experimental mice GFAPCre; *Rbm8a^f/+^* (N = 22, male = 12, female = 10) * indicates a *p*-value < 0.05, ** indicates a *p*-value < 0.01, *** indicates a *p*-value < 0.001.

While the OFT can provide some indications for anxiety-like behaviors, the EPM test takes advantage of the rodent’s natural aversion to heights and open area versus their curiosity to explore novel environments and is more specifically geared towards measuring anxiety-like behaviors. To further explore the anxiety-like phenotype, we examined control and GFAPCre; *Rbm8a^f/+^* mice in the EPM test (Figure 3). Based on native mouse behavior, it is expected that an abnormal interaction or affinity to the open arms may be indicative of an altered anxiety state [42]. We first examined the total arm entry (Figure 3A,B) and travel distance (Figure 3C). We detected similar hyperactivity only in female GFAPCre; *Rbm8a^f/+^* mice in both the total entry (*p* = 0.045, Figure 3B) and travel distance (*p* = 0.017, Figure 3C). Additionally, only female (*p* = 0.044) experimental mice demonstrate a decreased cumulative duration immobile (Supplemental Figure S2A). Interestingly, both male and female GFAPCre;*Rbm8a^f/+^* mice traveled significantly longer distances in the open arms (male *p* = 0.018, female *p* = 0.009, Figure 3D), spent significantly more time in the open arms (male: *p* = 0.033, female: *p* = 0.013, Figure 3G), spent shorter immobile time in the closed arms (male: *p* = 0.03, female: *p* = 0.018), and spent longer immobile time in the open arms (male: *p* = 0.046, female: *p* = 0.003) than control mice did (Supplemental Figure S2B).

We secondly examined the entry frequency, the percentage of the entry, cumulative time, the percentage of time spent, and latency to open arms. Interestingly, although both male and female GFAPCre; *Rbm8a^f/+^* mice travel longer distance and spent more time in the open arms compared to control mice (Figure 3D,G), only female (*p* = 0.007) but not male GFAPCre; *Rbm8a^f/+^* mice showed significantly increased entries to open arms (Figure 3E). Importantly, this difference became more significant when we measured the percentage of open arm entry (*p* = 0.0007, Figure 3F). Consistently, only female GFAPCre; *Rbm8a^f/+^* mice (22.45 ± 4.14%) spent almost a two-fold percentage of time in open arms compared to control mice (14.65 ± 2.32%, *p* = 0.0066, Figure 3H). A longer latency to enter the open arms is generally interpreted as an indicator of higher anxiety or fear. Conversely, a shorter latency to enter the open arms suggests lower anxiety or fear. Interestingly, male (*p* = 0.010) but not female mice demonstrated a significantly decreased latency to open arms (Figure 3I), suggesting that sex may differentially impact how anxiety is presented.

Thirdly, although we did not detect significant changes of total entry number in the closed arms in different groups (Figure 3J), only female (*p* = 0.0007) GFAPCre; *Rbm8a^f/+^* mice (66.11 ± 4.49%) showed a significant reduction in percentage of entry number in closed arms, compared to control mice (74.56 ± 3.73%, Figure 3K). Consistently, female (*p* = 0.033) but not male GFAPCre; *Rbm8a^f/+^* mice spent significantly less time in the closed arms (Figure 3L). For both sexes, experimental mice did not demonstrate any significant differences in the latency to closed arms (Figure 3N). Moreover, neither male nor female mice show a significant difference between genotype groups in the entry number in center, cumulative duration in center, or latency to center (Supplemental Figure S3A–C).

Increased entry and time spent in the open arms and decreased percentage entry and time spent in the closed arms of the EPM test together suggest diminished anxiety-like states in GFAPCre; *Rbm8a^f/+^* mice. Overall, the results show clear indications of decreased anxiety-like behavior, especially in female GFAPCre; *Rbm8a^f/+^* mice relative to controls.

### 3.4. Motor Coordination Is Unaffected in GFAPCre; Rbm8a^f/+^ Mice

**Figure 4:**
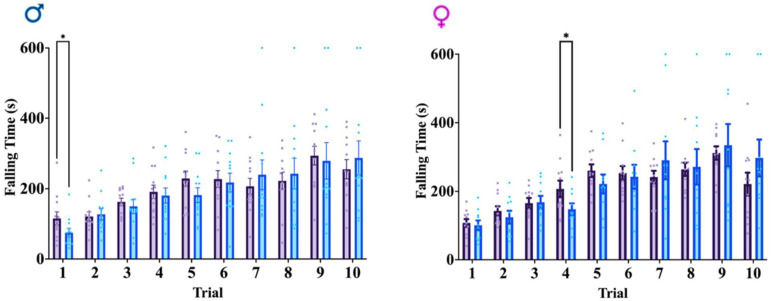
Rotarod test results across genotypes and sexes. Average falling time across ten total trials. Data shown are mean +/− SEM for control mice *Rbm8a^f/+^* (N = 21, male = 12, female = 11) and experimental mice GFAPCre; *Rbm8a^f/+^* (N = 22, male = 12, female = 10) where * indicates a *p*-value < 0.05.

As we detected hyperactivity in our mice, we wanted to explore the possibility that motor coordination could potentially contribute to or confound the interpretation of hyperactivity. To do this, we performed the rotarod test, which has been particularly attuned to detecting dysfunction of the cerebellar function underlying this motor function [41]. Rotarod test in both males and females demonstrates no consistent significant differences between groups in falling time, although select trials do indicate some degree of variance (Figure 4). Given the lack of overt significant differences in falling time in the rotarod test, the results of motor function behaviors support a hyperactive phenotype in the GFAPCre; *Rbm8a^f/+^* mice, across sexes.

### 3.5. Other Defects in GFAPCre; Rbm8a^f/+^ Mice

**Figure 5:**
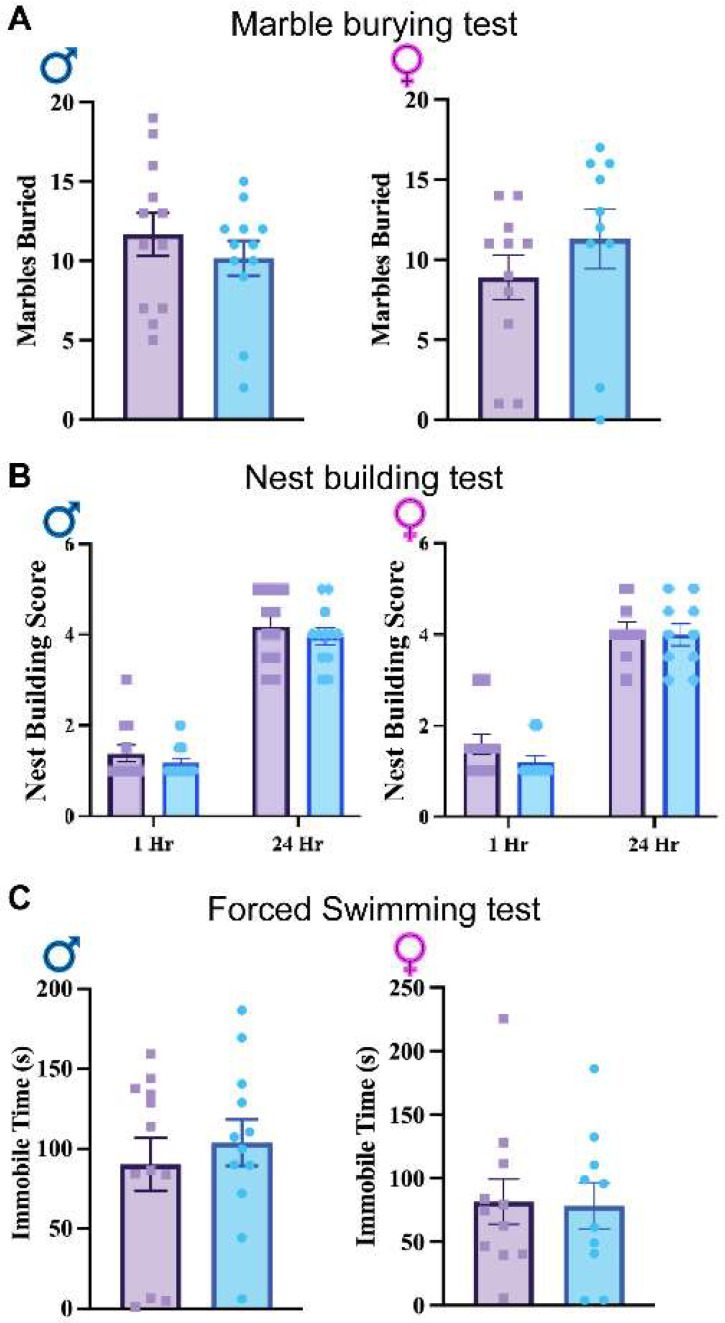
(**A**). Results of the average number of marbles buried in the marble burying test. (**B**) Nest building test scores across genotypes and sexes at 1 and 24 h. (**C**) Tail suspension test immobile times across genotypes and sexes. Data shown are mean +/− SEM for control mice *Rbm8a^f/+^* (N = 21, male = 12, female = 11) and experimental mice GFAPCre; *Rbm8a^f/+^* (N = 22, male = 12, female = 10).

To further examine other potential alterations, marble burying (Figure 5A), nest building (Figure 5B), and tail suspension tests (Figure 5C) were performed. Neither the marble burying nor nest building tests demonstrated significant differences in obsessive or repetitive behaviors between groups across sex, indicating a lack of difference in these behaviors (Figure 5A,B). The tail suspension test also failed to elicit a significant difference between groups across sex, indicating there is no depressive-like effect between GFAPCre; *Rbm8a^f/+^* and *Rbm8a*^*f*/+^mice (Figure 5C). These data suggest RBM8A in astrocytes does not affect depressive-like behaviors.

Recently, astrocytes in different regions of the brain, including the hippocampus, have been shown to play critical roles in behavioral regulation and diseases [44,45,46,47]. To determine the effect of *Rbm8a* haploinsufficiency in astrocytes, we sought to determine GFAP-positive astrocyte density in the hippocampus (Supplemental Figure S4). Interestingly, we detected a higher astrocyte population in the hippocampus of female GFAPCre; *Rbm8a^f/+^* mice, but not in males (Supplemental Figure S4C,D). These data, again, support a sex-dependent role of RBM8A in the regulation of astrocyte density that may correlate to the sex-specific behavioral changes observed above.

## 4. Discussion

Here, we have investigated the role of *Rbm8a*, a core member of the EJC, in GFAP-positive astrocytes using conditional *Rbm8a* heterozygous knockout mice (*GFAPCre*; *Rbm8a^f/+^*). We validated the mouse line by confirming the decreased RBM8A expression in GFAP-expressing cells using RT-PCR and Sanger sequencing, qRT-PCR, immunohistochemistry, and Western blot. Additionally, we performed several behavioral tests with these mice to examine the potential altered anxiety-related locomotion behaviors.

We observed half levels of RBM8A in *GFAPCre*; *Rbm8a^f/+^* mice, even though the deletion only happens in GFAP-positive cells. Several reasons could explain this significant change. First, astrocytes are the most abundant cells in the mouse brain. Even though there are other unaffected neurons expressing higher levels of the RBM8A protein, the level in neurons may not compensate for the cell number. Second, immunostaining detects protein levels that do not exactly correlate with mRNA levels, as many studies have demonstrated [48,49,50]. Third, the exon junction complex factors, including RBM8A, are required for transcription [51], protein translation [52,53,54], and mRNA stability [55], which may have feedback regulation on its own expression. Fourth, the decrease in RBM8A in astrocytes may exert cell non-autonomous effects on the surrounding cells. Fifth, the deletion can occur in adult GFAP-expressing neural stem cells and their progeny in the brain. These possible reasons may explain why we observed half RBM8A levels in GFAPCre; *Rbm8a^f/+^* mice despite the loss of one copy of *Rbm8a* gene only in astrocytes.

The behavioral results demonstrated that these mice show an increase in locomotion function and general activity, pointing to a hyperactive phenotype in both male and female GFAPCre; *Rbm8a^f/+^* mice. Additionally, the elevated plus maze test indicates that these experimental mice may display decreased anxiety behaviors, relative to control mice. Finally, the other tests did not reveal any significant impact on obsessive, repetitive, or depressive behaviors in the experimental mice, relative to control mice.

The role of glial cells in neural disorders has recently become a growing point of research. Astrocytes are known to be in close communication with neurons during development and astrocytes, in particular, are able to engage in bi-directional signaling with up to 100,000 synapses [28]. Astrocytes express tremendous functional and proteomic diversity based on their specialization in the neural circuit of interest [29]. Particularly, astrocytes have been shown to facilitate synapse elimination [46,56]. Our findings provide new insights into how *Rbm8a* gene mutations in the astrocytes can work as a risk factor for neurodevelopmental diseases. Analyses of human ASD brains post mortem demonstrate increases in glial reactivity and, thus, it is possible that the dysregulation of an astrocyte-mediated inflammatory response may be implicated in the generation of ASD brains [28]. The RNAseq result in our previous study [57] detected an upregulation of GFAP mRNA in the *Nes-cre-Rbm8a ^f/+^* brain at P17. Consistently, this study reveals that *Rbm8a* heterozygous KO in GFAP-positive astrocytes leads to a sex-specific increased cell astrocyte density in the hippocampus. However, as GFAP-cre is also expressed in adult NPCs [58], which could delete *Rbm8a* in adult NPCs and their progenies, the current study cannot completely exclude the potential contribution of postnatal defects from adult NPCs. Future studies will be required to further delineate the underlying pathophysiological mechanisms. It would be interesting to further narrow down if the *Rbm8a* deletion from adult NPCs or astrocyte subtypes in different brain regions will mediate particular phenotypes. Such studies will provide a better understanding of circuitry mechanism for ADHD-like neurological diseases.

Our study indicates that *Rbm8a* heterozygous KO specifically in postnatal GFAP-expressing astrocytes is sufficient to modify behaviors leading to hyperlocomotion. Hyperactivity has been defined as an increased locomotion activity and spontaneous exploration indicated by variables such as distance traveled, moving duration, mobility duration, and average velocity [59,60,61]. Mouse models where hyperactivity is found often also explore aspects of attention or impulse control in the context of ADHD. Human ADHD is characterized by a dysfunction in activity, attention, and impulse control. Mouse models of ADHD are heterogeneous and diverse, thereby substantiating evidence towards the polygenic nature of ADHD epidemiology [62].

It is also known that elements of attention, impulsivity, and hyperactivity could be associated with ASDs, with nearly half of the ASD population meeting the diagnostic criteria for ADHD, as stipulated in the DSM-V [63,64]. Both ASDs and ADHD have been shown to carry a significant genetic basis, with findings of over eight in ten ASD patients exhibiting identifiable genetic variants in one or numerous neural and non-neural genes [65]. It has been shown that *RBM8A* and other genes located in the 1q21.1 copy number variant are associated with neurodevelopmental and neurodegenerative disease, and especially with ASDs and ID [37,66]. Therefore, finding that a hyperactive phenotype manifests after the conditional deletion of *Rbm8a* in the mouse astrocytes may demonstrate a potential link to ASDs and ADHD worthy of further insight.

Consistent with this notion, there are several mouse models of ADHD and ASDs, which display hyperactivity in the mouse behavioral profile. ADHD often presents alongside generalized anxiety disorders, adding diagnostic complexity to the individual or dual diagnoses [67]. Similarly, anxiety is often a major co-occurring symptom in ASD patients [68]. However, in both neurodevelopmental disorders, more work is required to determine if anxiety is an independent (comorbid) disorder or part of those disorders. Interestingly, our mice exhibit altered locomotion activity, suggesting hyperactivity and high anxiety are not always associated. Consistently, instead of an increased anxiety level in experimental mice, it was found that different models with hyperactivity may demonstrate a decreased anxiety, dependent on different stress conditions and genetics [69,70]. For example, histone acetyltransferase CREB binding protein (CBP) mutation causes Rubinstein–Taybi Syndrome (RTS), where dysfunction in chromatin or DNA function emerges as a critical component of ASDs. Knockout of the CH1 domain of CBP results in hyperactivity, reduced anxiety, and a disruption in synaptic homeostasis [71]. The Fragile Mental Retardation 1 locus (FMR1) is located on the X chromosome, and an expansion of triplet repeats preventing the proper production of the FMRP RNA-binding protein is the most common inherited pattern of mental retardation. FMRP modulates mRNA trafficking, dendritic maturation, and synaptic plasticity. Notably, FMR1 KO mice exhibit hyperactivity and also demonstrate decreased anxiety [72].

Multiple neurotransmissions have been implicated in hyperactivity, such as dopamine and GABA. Dopamine transporter-1 (DAT-1) is a re-uptake transporter of dopamine from the synaptic cleft back into the synaptic bouton of the presynaptic neuron. Its KO results in both hyperactivity and impulsivity in mice [73,74]. Among others, these studies contribute to growing evidence that GABA signaling and networks are vulnerable or contributory to the pathogenesis of ADHD [75,76,77]. CNTNAP2—the largest gene in the genome—regulates the neuron–glia interaction during brain development and contributes to the development of neuron axon substructures. The deletion of the Contactin-associated protein-like 2 gene (CNTNAP2) in mice also results in hyperactivity, alongside deficits in cortical projection, neuron migration, and a reduction in the number of GABAergic interneurons [78,79]. Consistently, our previous studies have revealed that *Rbm8a* plays a critical role in interneuron differentiation [24] and regulates the genes involved in GABA synapse formation [57]. However, the potential of RBM8A to regulate GABAergic synapses through astrocytes and its subsequent role in inducing hyperactivity is an intriguing avenue for future research.

## 5. Conclusions

This study demonstrates that the conditional heterozygous knockout of *Rbm8a* in GFAP-positive astrocytes leads to increased movement and sex-specific behavioral changes in mice, indicating RBM8A’s critical role in neurodevelopment and astrocyte functions. Moreover, the alteration in astrocyte density underlines the significance of RBM8A in central nervous system pathology, offering new perspectives on neurodevelopmental disorders through astrocytic involvement.

## Data Availability

All Supplemental Data and Materials are available online and upon reasonable request.

## Supplementary Materials

The following are available online at https://www.mdpi.com/article/10.3390/cells13060498/s1, Figure S1: Western blot to confirm reduced RBM8A protein in GFAPCre; *Rbm8a^f/+^* mice and RBM8A protein is not changed in neurons. Figure S2: Immobile time in the EPM test. Figure S3: Total entries and duration in the center of the EPM test. Figure S4: Astrocyte density in the hippocampi of control and *GFAPCre*; *Rbm8a^f/+^* mice.
