# Supplementary material for: The Exon Junction Complex Factor RBM8A in Glial Fibrillary Acid Protein-Expressing Astrocytes Modulates Locomotion Behaviors
Source: Cells. 2024 Mar 13;13(6):498. doi: 10.3390/cells13060498 (PMC10968791; doi:10.3390/cells13060498)
Supplement: Supplementary file 1 [file cells-13-00498-s001.zip › cells-2610301-supplementary.pdf]

Supplemental Figures

The Exon Junction Complex Factor RBM8A in Glial Fibrillary Acid Protein-Expressing Astrocytes Modulates Locomotion Behaviors

Shravan Asthana [1,2,†], Jennifer Mott [1,†], Mabel Tong [1], Zifei Pei [1] and Yingwei Mao [1,*]

Supplemental S1

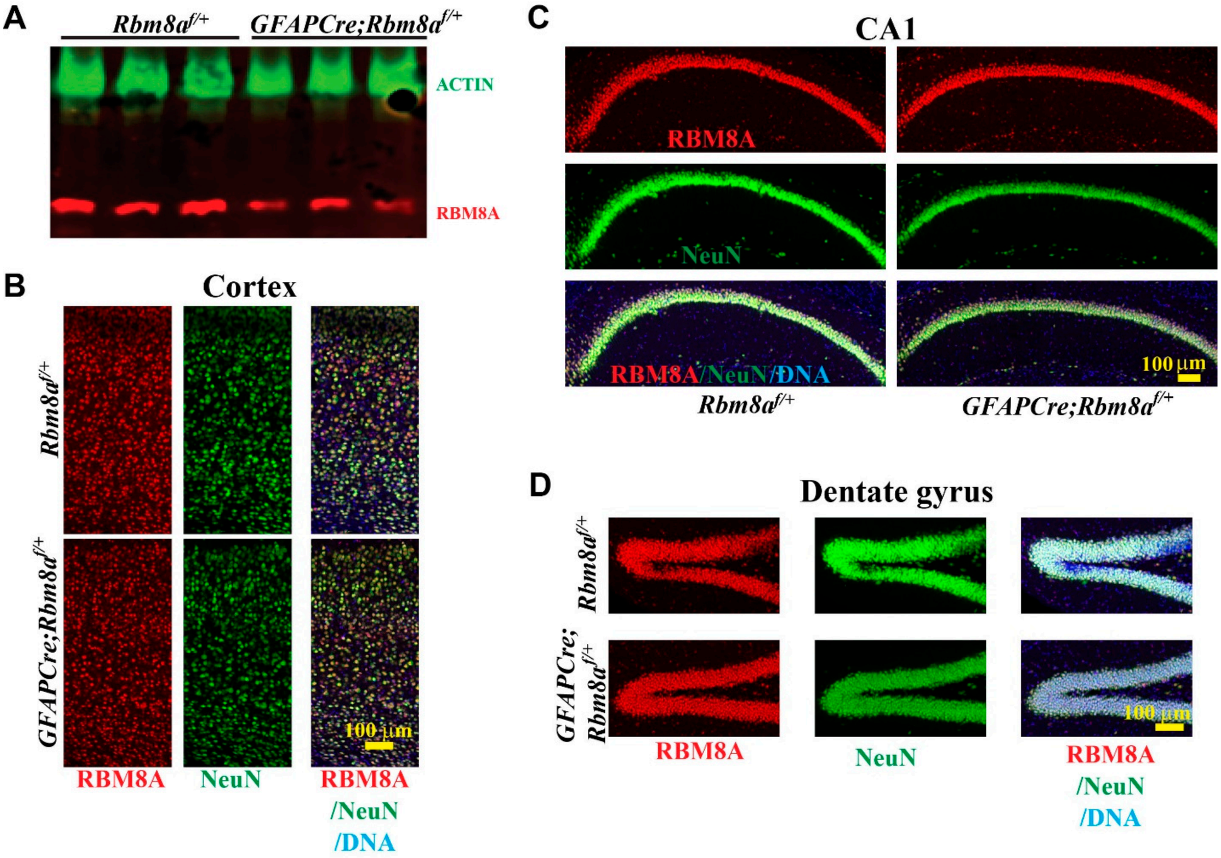

Figure S1. (A) Western blot of RBM8A (red) and ACTIN (green) proteins in control mice $Rbm8a^{f/+}$ and GFAPCre; $Rbm8a^{f/+}$ mice (n=3). (B) Immunostaining of NeuN(green) and RBM8A (red) in the cortex of control $Rbm8a^{f/+}$ and GFAPCre; $Rbm8a^{f/+}$ mice. (C)) Immunostaining of NeuN(green) and RBM8A (red) in the CA1 of control $Rbm8a^{f/+}$ and GFAPCre; $Rbm8a^{f/+}$ mice. (D) Immunostaining of NeuN(green) and RBM8A (red) in the dentate gyrus of control $Rbm8a^{f/+}$ and GFAPCre; $Rbm8a^{f/+}$ mice. Scale=100μm.

Supplemental S2

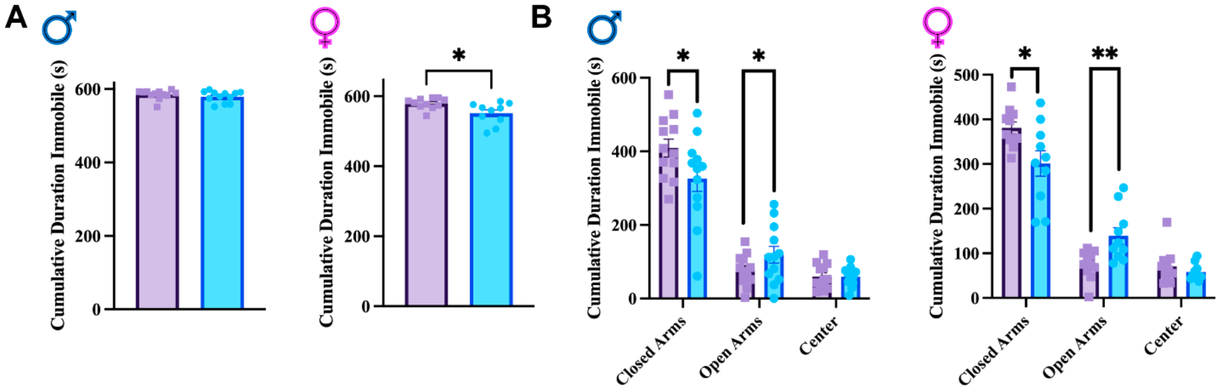

Figure S2. (A) Cumulative duration immobile in the EPM. **(B)** Cumulative duration immobile on the border and center of the EPM. Data shown are mean +/- SEM for control mice *Rbm8a*$^{f/+}$ (N = 21, male = 12, female = 11) and experimental mice GFAPCre; *Rbm8a*$^{f/+}$ (N = 22, male = 12, female = 10). * indicates a P-value < 0.05, ** indicates a P-value < 0.01.

Supplemental S3

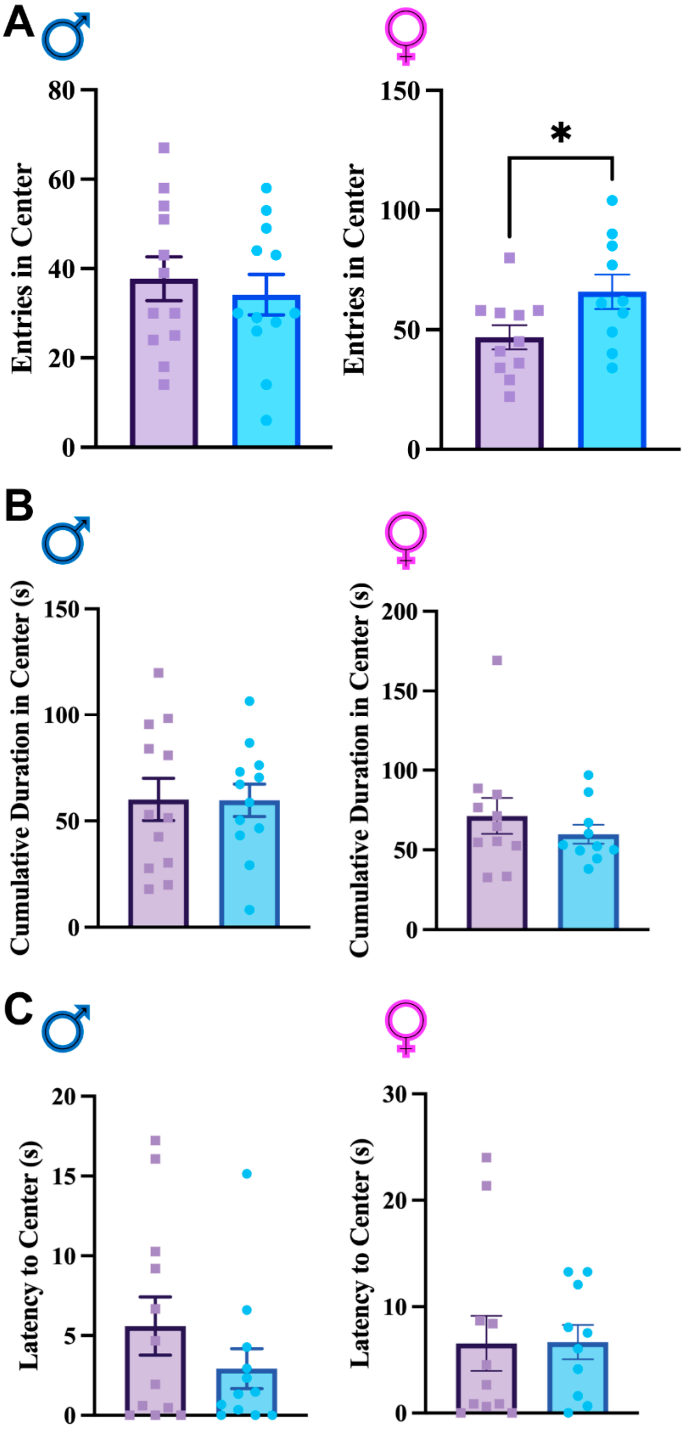

Figure S3. (A) Total entries in the center of the EPM. **(B)** Cumulative duration in the center of the EPM. **(C)** Latency to the center zone. Data shown are mean +/- SEM for control mice *Rbm8a*$^{f/+}$ (N = 21, male = 12, female = 11) and experimental mice GFAPCre; *Rbm8a*$^{f/+}$ (N = 22, male = 12, female = 10). * indicates a P-value < 0.05.

Supplemental S4

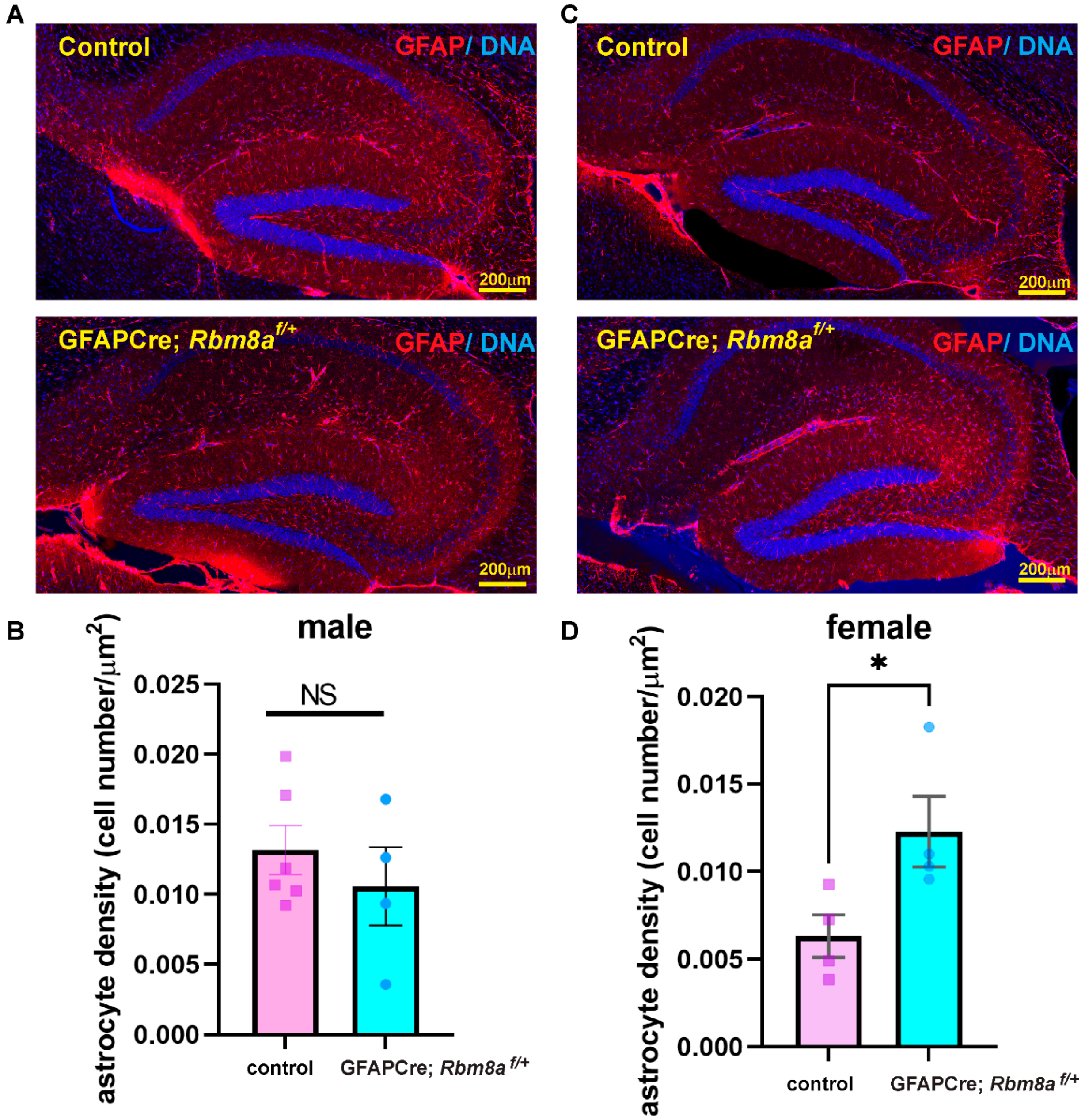

Figure S4. Astrocyte density in the hippocampi of control and *GFAPCre; Rbm8a*$^{f/+}$ mice. (A, B) No statistically significant difference was observed between astrocyte density in the hippocampi of male control and experimental mice. **(C, D)** Female experimental mice show significantly higher astrocyte density in the hippocampus than female control mice. Scale bar=200 μm. * indicates a P-value < 0.05.
